# Identification and characterization of ecdysis-related neuropeptides in the lone star tick *Amblyomma americanum*

**DOI:** 10.3389/fendo.2023.1256618

**Published:** 2023-08-25

**Authors:** Bo Lyu, Jingjing Li, Brigid Niemeyer, David Stanley, Qisheng Song

**Affiliations:** ^1^ Division of Plant Science and Technology, University of Missouri, Columbia, MO, United States; ^2^ Biological Control of Insect Research Laboratory, United States Department of Agriculture-Agricultural Research Station (USDA/ARS), Columbia, MO, United States

**Keywords:** *Amblyomma americanum*, tick, neuropeptides, ecdysis, bursicon

## Abstract

**Introduction:**

The lone star tick, *Amblyomma americanum*, is an important ectoparasite known for transmitting diseases to humans and animals. Ecdysis-related neuropeptides (ERNs) control behaviors crucial for arthropods to shed exoskeletons. However, ERN identification and characterization in *A. americanum* remain incomplete.

**Methods:**

We investigated ERNs in *A. americanum*, assessing their evolutionary relationships, protein properties, and functions. Phylogeny, sequence alignment, and domain structures of ERNs were analyzed. ERN functionality was explored using enrichment analysis, and developmental and tissue-specific ERN expression profiles were examined using qPCR and RNAi experiments.

**Results and discussion:**

The study shows that ERN catalogs (i.e., eclosion hormone, corazonin, and bursicon) are found in most arachnids, and these ERNs in A. americanum have high evolutionary relatedness with other tick species. Protein modeling analysis indicates that ERNs primarily consist of secondary structures and protein stabilizing forces (i.e., hydrophobic clusters, hydrogen bond networks, and salt bridges). Gene functional analysis shows that ENRs are involved in many ecdysis-related functions, including ecdysis-triggering hormone activity, neuropeptide signaling pathway, and corazonin receptor binding. Bursicon proteins have functions in chitin binding and G protein-coupled receptor activity and strong interactions with leucine-rich repeat-containing G-protein coupled receptor 5. ERNs were expressed in higher levels in newly molted adults and synganglia. RNAi-mediated knockdown of burs α and burs β expression led to a significant decrease in the expression of an antimicrobial peptide, defensin, suggesting they might act in signaling or regulatory pathways that control the expression of immune-related genes. Arthropods are vulnerable immediately after molting because new cuticles are soft and susceptible to injury and pathogen infections. Bursicon homodimers act in prophylactic immunity during this vulnerable period by increasing the synthesis of transcripts encoding antimicrobial peptides to protect them from microbial invasion. Collectively, the expression pattern and characterization of ERNs in this study contribute to a deeper understanding of the physiological processes in *A. americanum*.

## Introduction

Ticks are ectoparasitic arachnids that feed on the blood of animals, including mammals, birds, reptiles, and humans. They transmit a large number of pathogens responsible for diseases such as Lyme disease, Ehrlichiosis, and Rocky Mountain spotted fever (1, 2). Tick life cycles include four stages: egg, larva, nymph, and adult. Post-egg stage ticks attach and feed on a host at each stage before molting into the next stage (3). Their life cycles can last months to years, depending on the tick species and environmental conditions. Most research into tick biology is meant to improve or create novel tick control technologies.

The lone star tick, *Amblyomma americanum*, is common in the eastern United States and part of the Midwest (4), although most tick species are undergoing climate-driven range expansions (5). *A. americanum* feeds on various host species, such as deer, rodents, birds, and humans. The lone star tick transmits various diseases to human beings, including tularemia, southern tick-associated rash illness, and Rocky Mountain spotted fever. Like all ticks, *A. americanum* molts by shedding its old exocuticles and forming new ones. In arthropods, molting hormone (mainly 20-hyddroxyecdysone - 20E) activates enzymes that dissolve the old endocuticle (6). Animal nervous systems produce, store, and release neuropeptides (7, 8) that act as neurotransmitters in arthropods, to signal physiological and metabolic processes (9, 10). Plants also release peptides that act in cell-cell signaling in growth and defense (11). Coordinating physiological and behavioral changes during arthropod ecdysis is facilitated by ecdysis-related neuropeptides (ERNs), including eclosion hormone (EH), corazonin (Crz), ecdysis triggering hormone (ETH), myoinhibitory peptide (MIP), crustacean cardioactive peptide (CCAP), and bursicon (12, 13). These ERNs collectively contribute to regulating various aspects of the ecdysis process, such as initiation, modulation of behaviors, muscle activity, cardiac regulation, stress responses, and cuticle hardening. For example, EH acts in initiating the shedding of the exoskeleton, necessary for the growth and development of arthropods. During molting, Crz operates on the heart and other muscles to increase their contraction rates, which helps to enhance blood flow and ecdysis (14, 15). Immediately after ecdysis, bursicon activates tyrosine hydroxylase by phosphorylation, which in turn converts amino acid, tyrosine, into dihydroxyphenylalanine (DOPA) (16). DOPA-derived tanning agents, N-acetyldopamine (NADA) and N-β-alanyldopamine (NBAD), cross-link and harden the outer component of the procuticle. The inner portion forms the endocuticle. The cuticle then hardens and becomes more hydrophobic (17). Aside from actions in molting, bursicon homodimers act in prophylactic immunity during molts by increasing the synthesis of transcripts encoding antimicrobial peptides (AMPs). This activation occurs immediately after ecdysis when new cuticles are soft and susceptible to injury and pathogen infections (18, 19). The receptors for these ERNs have been identified in tick species, including MIP and GYRKPPFNGSIFamide (SIFamide) receptors in *Ixodes scapularis*, CAP2b (periviscerokinin) receptor and G protein-coupled receptor super-family in *Rhipicephalus microplus* (20–23). Proteomic and peptidomics analyses of the identified precursors led to the characterization of over 80 distinct Ixodoidea neuropeptides and receptors, including Crz, crustacean hyperglycemic hormone/ion transport peptide, proctolin, SIFamide, and tachykinin-related peptide (24–26). Overall, understanding of tick physiology is rapidly growing with research into tick neuropeptides and their receptors.

In this study, we conducted an analysis of ERN gene families in *A. americanum*. Our analysis generated information necessary to pose the hypothesis that appropriate RNAi constructs inhibit the expression of genes encoding both bursicon subunits and the AMP, defensin. Here, we report on our analysis of tick ERN gene families and the outcomes of experiments designed to test our hypothesis.

## Materials and methods

### Tick materials

The lone star ticks were acquired from Boehringer Ingelheim Vetmedica, Inc. (Fulton, MO, USA) and maintained under controlled laboratory conditions (27 ± 1°C, 80–90% relative humidity, and a 16:8 h light:dark cycle). Ticks are laboratory reared on contained animal systems approved by site IACUC and stored in clean vials between required feedings.

To identify the ERNs and antimicrobial peptides (AMP) induced by ERNs (such as burs α and burs β), newly emerged and unfed female ticks were subjected to a common gram-negative bacteria *Escherichia coli* (50 nL, 10^7^ CFU, 25 ± 1°C) (DH5 α strain, Thermo Fisher Scientific Inc., USA) for 3, 6, 12, and 24 h, respectively. The control groups were injected with phosphate buffer saline (PBS, 10 mM, pH 7.2) (137 mM NaCl, 2.7 mM KCl, 10 mM Na_2_HPO_4_, and 1.8 mM KH_2_PO_4_, pH 7.4). *E. coli* or PBS solution (50 nL) was injected using a Nanoject II AutoNanoliter Injector (Drummond Scientific Co., Broomall, PA, USA) fitted with a 3.5-inch glass capillary tube pulled by a needle puller (Model P-2000, Sutter Instruments Co., Novato, CA, USA) into the ventral side near the anus of ticks under a Leica M205 C stereomicroscope (Leica Microsystems, Wetzlar, Germany). Tick samples were homogenized in 500 µL of TRIzol using a homogenizer and stored at -80°C until further analysis. A total of 72 ticks were collected from 4 time points indicated above and pooled into 24 groups. Three biological replicates for each treatment were performed, each with three ticks, for RNA-seq expression profiling.

### cDNA library preparation and quality control

Total RNA was extracted from tick samples using the TRIzol reagent (Invitrogen, CA, USA). The RNA content was measured using the Qubit® RNA Assay Kit (Life Technologies, CA, USA), and RNA purity was assessed using an Implen NanoPhotometer® spectrophotometer (Westlake Village, CA, USA). The Agilent Bioanalyzer 2100 system and the RNA Nano 6000 Assay Kit (Agilent Technologies, CA, USA) were used to measure the integrity of the RNA. To prepare cDNA libraries, the NEBNext® UltraTM RNA Library Prep Kit from NEB (Ipswich, MA) was used with 1.5 μg RNA. The first and second strands were synthesized using M-MuLV Reverse Transcriptase, random hexamer primer, DNA Polymerase I, and RNase H (Beckman Coulter, Beverly, USA). Real-time PCR and Qubit were used to quantify the library (Thermo Scientific, MA, USA), and an Agilent 2100 Bioanalyzer (Santa Clara, CA) was used to find the size distribution. The fastq formatted raw data was processed using custom-written perl scripts. To generate clean reads, reads with adapters, ploy-N, and low quality were eliminated from the raw data. Raw data (in fastq format) was processed using in-house perl scripts to produce clean reads. The process involved removing reads with adapters, poly-N sequences, and low quality reads from the raw data. Metrics such as Q20, Q30, GC-content, and sequence duplication level were calculated for the clean data (27). All subsequent analyses were conducted using the high-quality clean data. These data were used as the references for all subsequent studies. The sequenced data were deposited in the Sequence Read Archive (https://www.ncbi.nlm.nih.gov/genbank/) with the accession number PRJNA982785.

### Identification and annotation of ERN genes

The Pfam database (https://www.ebi.ac.uk/interpro/) and the HMMER 3.0 program (http://hmmer.janelia.org/) were used to find potential ERNs. Canonical conserved domains were used to identify the EH (a predicted mature peptide has six cysteine residues)(28), Crz (a predicted mature peptide is composed of 11 conserved amino acids pGln-Thr-Phe-Gln-Tyr-Ser-Arg-Gly-Trp-Thr-Asn) (29), burs α and burs β (bursicon subunits have 11 conserved cysteine residues) sequences (30). The NCBI Conserved Domains Database (http://www.ncbi.nlm.nih.gov/cdd) annotation was used to validate whether the candidate ERNs members contained the protein domain.

### Evolutionary relatedness and phylogenetic analysis

The CLANS program performed cluster analyses on the ERN candidate sequences and their previously annotated orthologs (31). For 50,000 cycles, the input datasets were clustered using local blastp with a default *e*-value of 10 and a *p*-value of 10e^-4^. After isolating and disconnecting sequences, the generated 3D maps were simplified into 2D for improved visualization. The accessible NCBI and Swiss-Prot protein databases were searched for ERN sequences from other arachnids. The ClustalW2 tool was used to perform multiple sequence alignments. Phylogenetic trees of the conserved domain sequences of two groups of arachnids, Araneae (*Caerostris darwini*, *Nephila pilipes*, *Argiope bruennichi*, *Trichonephila clavipes*, *T. inaurata*, *C. extrusa*, *Parasteatoda tepidariorum*, and *Araneus ventricosus*) and Ixodida (*Ixodes scapularis*, *A. americanum*, *Rhipicephalus microplus*, *R. sanguineus*, *Dermacentor variabilis*, and *D. silvarum*), were constructed to examine the phylogeny in the ERN sequences (**Table S1**). The neighbor-joining (NJ) method with the bootstrap (1,000 replicates, Poisson mode, and pairwise deletion) was used to construct phylogenetic trees using MEGA11 (32). ITOL (https://itol.embl.de/) was used to beautify the phylogenetic tree and present supported values.

### Protein characteristics, functional domain, and structural modeling

The ERNs’ and their *A. americanum* protein ortholog properties, including 2D and 3D conformations, were illustrated to gain insights into their structural characteristics and potential functional implications. The putative ERNs’ theoretical isoelectric point (pI) and molecular weight (MW) were calculated using online toolkits (http://expasy.org/tools/). Jalview software (http://www.jalview.org/) was used to visualize the ERNs amino acid alignment. WebLogo for multiple sequence analysis was used to build the sequence logo (http://weblogo.berkeley.edu/). PSIPRED (http://bioinf.cs.ucl.ac.uk/psipred/) and SWISS-MODEL (https://www.swissmodel.expasy.org/) were used for secondary structure predictions. I-TASSER (https://zhanggroup.org/I-TASSER/) was used to generate *de novo* models of ERNs proteins following the C-score and TM score. ProteinTools was used to evaluate the predicted 3D protein structure and detect structural characteristics, including salt bridges, hydrogen bond networks, and hydrophobic clusters (https://proteintools.uni-bayreuth.de/).

### Gene structure, functional annotation, and regulatory network

The complete coding sequences of ERNs from tick species were obtained from the NCBI database (**Table S2**). ERN sequence domain motifs were shown using the Multiple Em for Motif Elicitation (MEME) tool (http://meme-suite.org/tools/meme). The Blast2GO software (v. b2g4pipe_v2.5) was used to functionally annotate the ERN genes, with an *e*-value of 1e^-6^. The R program “GOplot” was used to depict the functional annotations of the discovered ERNs genes from three GO types: biological process, molecular function, and cellular component. Based on the RNA-seq data, the String database (https://string-db.org/) was used to find interactive nodes and edges. Protein-to-protein interaction (PPI) data with a string score > 400 were used to build the entire network, and Cytoscape (https://cytoscape.org/) was used to visualize the constructed network.

### Spatial and temporal gene expression sample preparation

The midguts, synganglia, and salivary glands were isolated from 20 female adults to analyze tissue-specific transcript levels encoding EH, Crz, burs α and burs β. Adult ticks were transferred into a sterile dissecting dish containing 1X PBS (pH 7.4) supplemented with 1 unit/μL of RNase inhibitor. We used forceps and sterile disposable scalpels to remove the exoskeleton under a dissecting microscope (Wild Heerbrugg WILD M5A Stereo Scope, Adlon Instruments Inc., St. Louis, MO, USA) to expose selected areas. Fifty eggs, 30 zero h larvae and three individuals for each of the replete nymphs, 0 h female adults and 24 h female adults were collected for analysis. Three biologically independent replicates were conducted for each treatment.

### Preparation and microinjection of double-strained RNA

The two bursicon subunits act in CNS-mobilized prophylactic immunity by stimulating AMP expression during molts (18, 19). We used RNAi knockdowns of the genes encoding the two subunits to determine the effects on defensin expression (the main type of AMP in ticks). dsRNA templates for GFP, burs α, and burs β were synthesized by PCR, using gene-specific primers containing the T7 polymerase promoter sequence at their 5′ ends (**Table S3**). The T7 templates for dsRNA synthesis were generated using the PCR-amplified target sequences as templates. The dsRNAs, ranging from 38 to 40 base pairs (bp), were designed based on the open reading frame (ORF) of the target transcripts. Prior to cDNA synthesis, genomic DNA (gDNA) was removed by using DNase1 (Thermo Fisher Scientific Inc., Waltham, MA, USA) to avoid potential interference from genomic contamination in the subsequent analysis. The resulting cDNA was purified using a PCR clean-up kit (M1001-50, EZ BioResearch LLC, St. Louis, MO, USA). dsRNA was synthesized from the purified PCR product using the HiScribe™ T7 Quick High Yield RNA Synthesis Kit (E2050, New England Biolabs Inc., Beverly, MA, USA) following the manufacturer’s instructions. dsRNAs were treated with DNaseI, purified using phenol/chloroform extraction and isopropanol precipitation, and dissolved in diethylpyrocarbonate-treated water (Life Science Products, Inc., Frederick, USA). The dsRNA concentration was determined on a Nanodrop 2000 spectrophotometer (Thermo Fisher Scientific Inc., Waltham, MA, USA), and its integrity was checked on 1% agarose gels. The dsRNA was stored at -80°C for later use.

Unfed zero h female adult ticks were injected with 1 μg/tick dsGFP, dsburs α, and dsburs β separately (7.5 μg/μL) into the ventral side near the anus using a Nanoject II Auto-Nanoliter Injector (Drummond Scientific Co., Broomall, PA, USA), equipped with a 3.5-inch glass capillary tube prepared on a needle puller (Model P-2000, Sutter Instruments Co., Novato, CA, USA). Ticks were maintained under standard conditions after injection.

### Quantitative real-time PCR validation

TRIzol reagent (Thermo Fisher Scientific Inc., MA, USA) was used to extract total RNA from ticks. The reverse transcription of 1 μg total RNA from each sample was performed using a High-Capacity cDNA Reverse Transcription Kit (Thermo Fisher Scientific Inc., USA). qRT-PCR reactions were run with a mixture of cDNA, forward and reverse primers, and iTaq™ Universal SYBR®Green Supermix (Bio-Rad Laboratories, CA, USA). The qRT-PCR program included one cycle of 95°C (3 min), followed by 45 cycles of 95°C (10 s) and 60°C (60 s), and then 35-40 cycles of 65-95°C (0.5°C increments at 2-5 sec/step). Melt curve analyses were conducted to verify reaction specificity. Primer efficiencies were calculated using standard curves generated from serial dilutions of cDNA samples. The reference gene V-ATPase subunit C (*VAC*) gene was used for normalization in the tissue/developmental profiling, as previous studies have shown it is stably expressed in *A. americanum* (33). The gene expression fold change was determined using the relative quantitative method (2^-△△CT^); the primers used are listed in **Table S3**.

### Statistics

Statistical analyses of qRT-PCR data and other parameters were performed using one-way analysis of variance (ANOVA) and Student’s t-test in GraphPad Prism (V.5.02, GraphPad Software, Inc). Adobe Photoshop CS6 and Adobe Illustrator CC (https://www.adobe.com/) were used to process and group figures.

## Results

### Arachnid ERN catalogs

**Figure 1:**
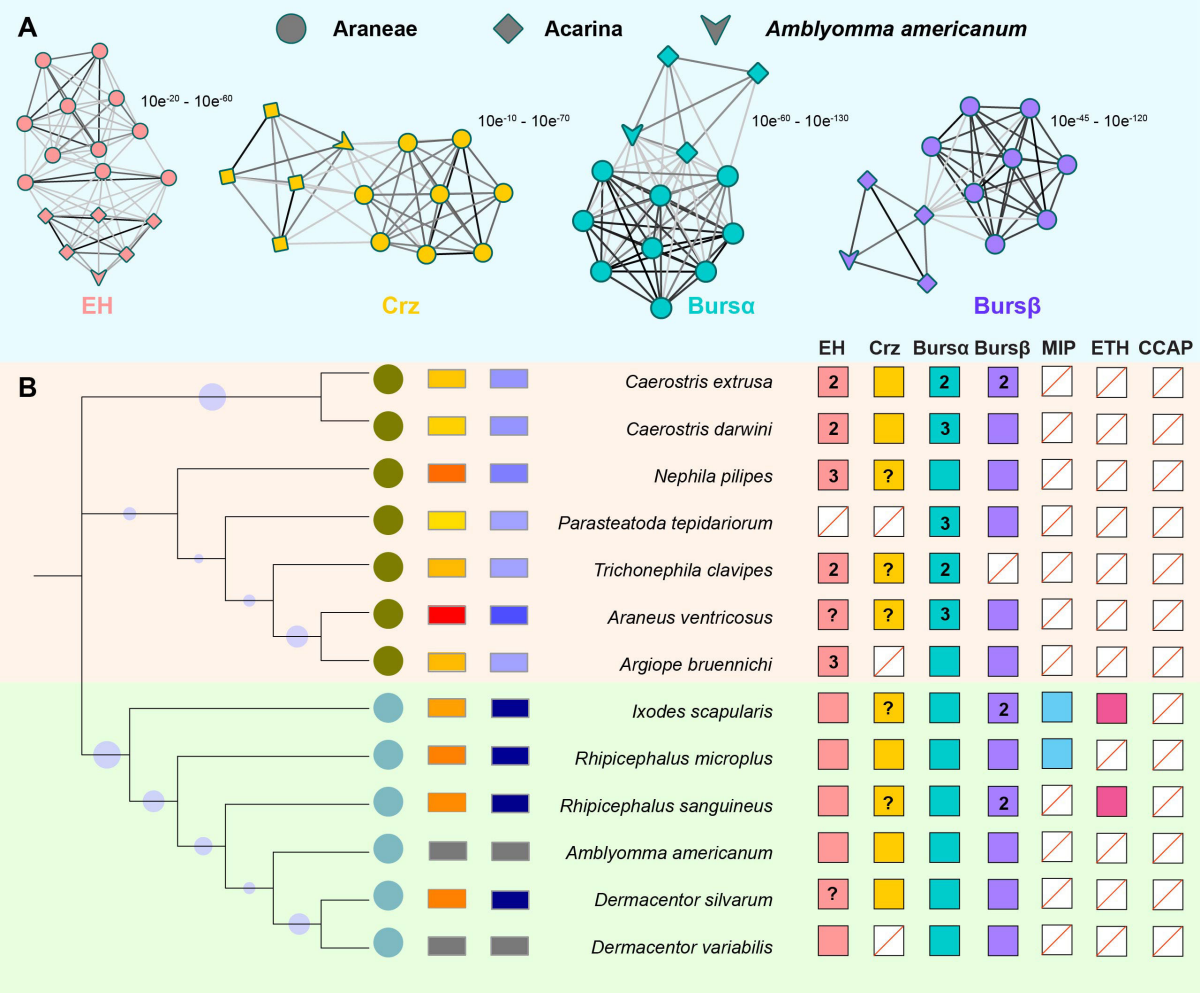
Ecdysis-related neuropeptide (ERN) catalogs in *A. americanum*. **(A)** 2D cluster maps created with the CLANS program showed the evolutionary relatedness between Araneae and Acarina ERNs. Lines with a *p*-value threshold correspond to BLAST connections. Different symbols (circles for spiders, squares for ticks) are used to distinguish the analyzed sequences, while different colors (e.g., blue and red) highlight the presence and distribution of the four ERNs. **(B)** ERNs were discovered for each species and displayed on a current phylogeny. Colored squares indicate the presence of ERNs. Empty squares show putative loss of the corresponding ERN with a red line. The ‘‘?’’ denotes the absence of a genome and the inability of transcriptome data to identify an ERN homolog. The total number of found paralogs for an ERN is given inside the appropriate square if more than one was discovered.

**Figure 2:**
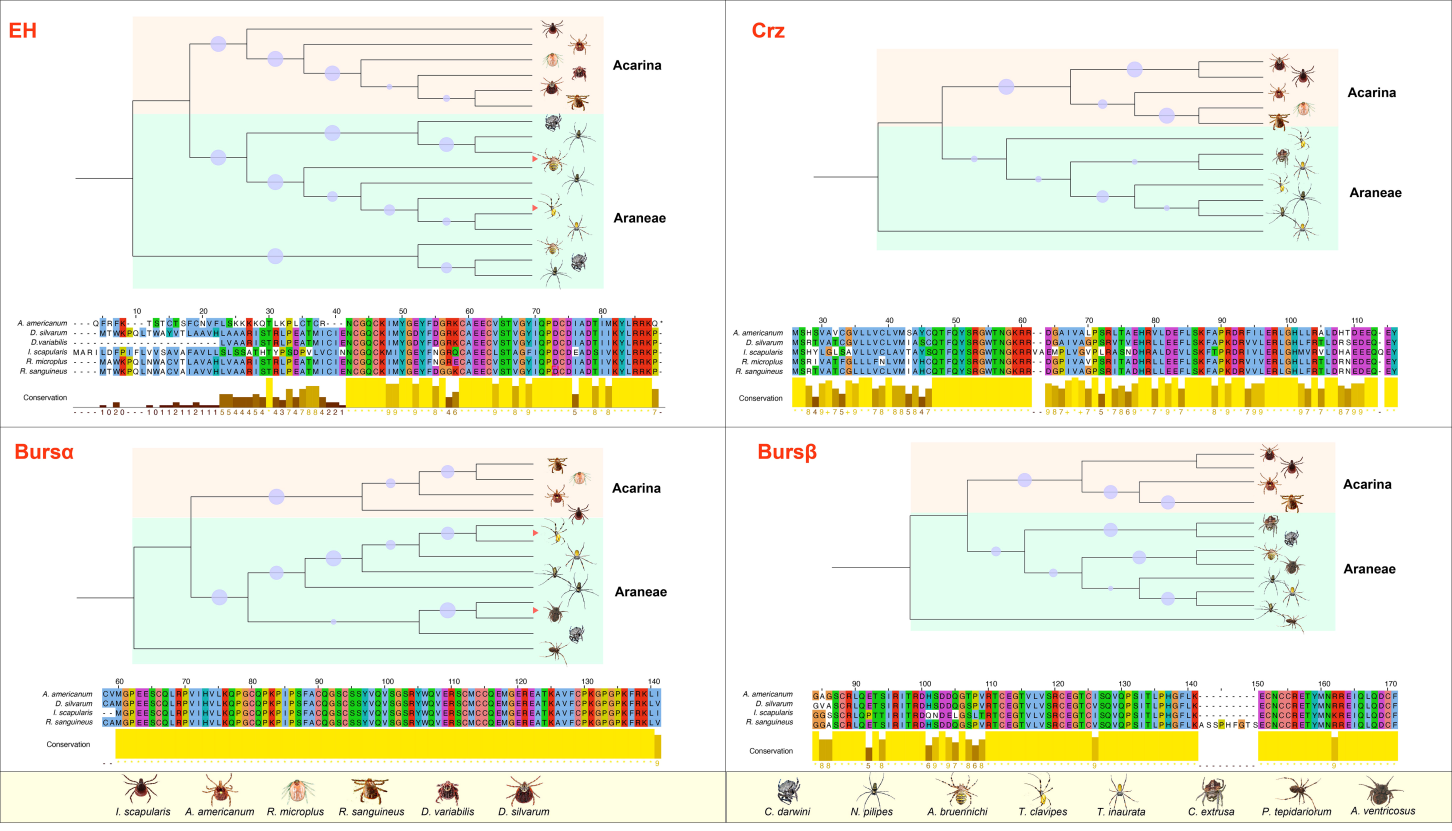
Phylogenetic analysis and amino acid alignment of four ERNs in *A. americanum*. The phylogenetic tree was created using MEGA11 by the NJ method analysis with 1000 repetitions utilizing the ERN gene sequences from Araneae and Acarina. The bootstraps from 50% to 100% were represented as dots in the phylogenetic tree. Jalview in Clustal format showed the amino acid alignment of the putative ERNs from Araneae and Acarina. Table S provides a list of ERNs employed in the sequence analysis.

ERNs are merged in arachnids (Araneae + Acarina; **Figure 1**), including seven spider species and six tick species with available COI sequences. EH, Crz, burs α, and burs β are highly related between Araneae and Acarina, and these *A. americanum* genes have conserved relatedness among tick species (**Figure 1A**). Arachnid species have one EH and one burs α sequence, whereas Araneae species have two or more corresponding sequences (**Figure 1B**). Two burs β sequences present in *I. scapularis* and *R. sanguineus.* Ticks have more complete ERN catalog annotations and fewer uncharacterized sequences than spiders. Burs α is present in all species investigated (**Figure 1**), and has the highest value compared with EH, Crz, and burs β. Except for Crz in *D. variabilis*. Other tick species have all four confirmed or putative ERNs. Nonetheless, the presence of three other ERNs (i.e., MIP, ETH, and CCAP), is less documented in arachnids (**Figure 1**). The evolutionary relationships among three *A. americanum* ERNs (EH, Crz, and burs α) have a closer evolutionary relationship with those of Araneae compared to other tick species. The conserved amino acid sequences of four ERNs were used to construct phylogenetic trees (**Figure 2**), and these ERNs were conserved within tick and spider species. Additionally, the evolutionary process of these ERNs in *A. americanum* might have involved selective pressure and adaptation that led to a more specialized branch within the phylogenetic tree (**Figure 2**).

### Protein properties and conserved ERN domains in *A. americanum*

We used 24 cDNA libraries to identify specific ERNs, including EH, Crz, burs α, and burs β based on the conserved domain motifs described previously (28–30). According to their *e* values (1.0*E* ^-20^), these sequences feature high NR similarity. We used online toolkits to estimate specific protein features of these four ERNs. According to subcellular localization prediction, these ERNs are associated with the extracellular matrix and plasma membrane (**Table S4**). No signal peptide occurs in the ERNs. The isoelectric points/molecular weights of EH, Crz, burs α, and burs β were 9.15/9,199.8, 8.00/12,771.8, 8.93/18,341.6, and 8.19/20,599.5, respectively. These values for the *A. americanum* ERNs were higher than the other three species, including *I. scapularis*, *R. sanguineus*, and *D. silvarum* (**Figure S1**).

### Structural modeling of four ERNs

**Figure 3:**
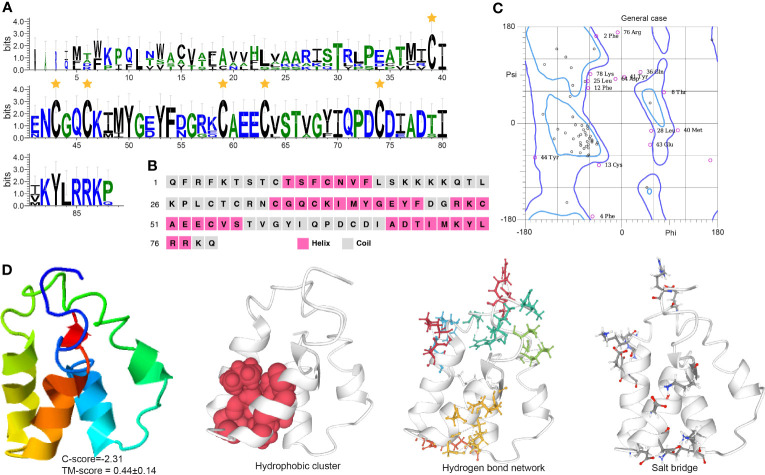
Functional domain and structural modeling of EH in *A. americanum*. **(A)** Sequence logo for the multiple sequence analysis of the EH protein. The stars indicate the conserved cysteine residues. **(B)** PSIPRED secondary structure prediction for EH protein. **(C)** Ramachandran plot analysis for the crystal structure of EH protein. The plot shows the distribution of phi and psi angles for each residue in the protein structure. **(D)** I-TASSER analysis of the protein structure for EH protein. The model consists of multiple domains with different colors representing different domains. The modeling protein structure was analyzed using ProteinTools to find hydrophobic clusters, hydrogen bond networks, and salt bridges for the EH protein.

The EH sequence has a conserved Pfam domain (PF04736) and a canonically predicted mature peptide containing six cysteine residues, Cys31, Cys34, Cys37, Cys50, Cys54, and Cys66 (**Figure 3A**). The six cysteine residues formed three disulfide bonds that may add protein stability to maintain the EH function. Secondary structure analysis indicates EH includes α-helices, which form repeating patterns of phi and psi angles and coiled structures (**Figures 3B, C**). Four helix regions and five coil regions occur in the protein (**Figure S2A**). The I-TASSER results indicate the predicted model was convincing with a -2.31 C-score (typically in the range of -5 to 2) and 0.44 ± 0.14 TM score (**Figure 3D**) (a TM-score < 0.17 means a random similarity). Functional cluster analyses indicate the protein includes a hydrophobic cluster (42.4 average residues), nine hydrogen bond networks (2.86 average distance and 153.51° average DHA), and six salt bridges (0.28 fraction of charged residues (FCR) and 0.31 kappa value (κ)).

**Figure 4:**
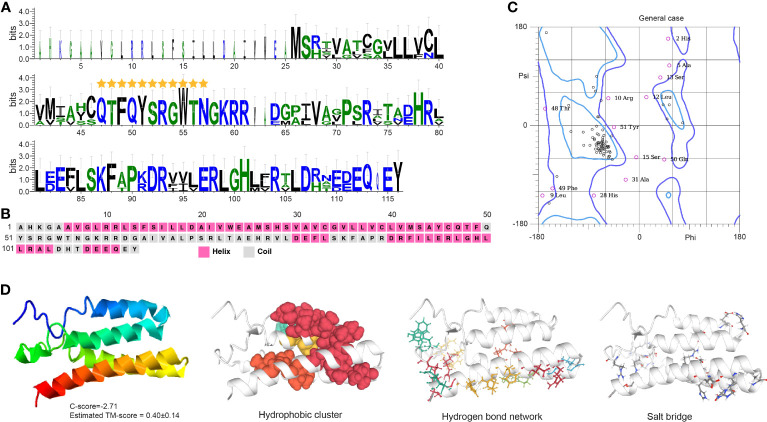
Functional domain and structural modeling of Crz in *A. americanum*. **(A)** Sequence logo analysis of the Crz protein. The sequence logo shows the conservation of amino acid residues at each position, with each letter’s height representing the conservation degree. The stars indicate the conserved domain QTFQYSRGWTN. **(B)** Secondary structure prediction of Crz protein sequence using PSIPRED. **(C)** Ramachandran plot analysis for the crystal structure of CrZ protein. The analysis indicates that most residues are in the allowed and generously allowed regions, suggesting the good quality of the protein structure. **(D)** I-TASSER analysis of the protein structure for Crz protein. The model consists of multiple domains with different colors representing different domains. The modeling protein structure was analyzed using ProteinTools to find hydrophobic clusters, hydrogen bond networks, and salt bridges for the EH protein.

Crz contains a typical PF17308 domain with a mature peptide made of 11 highly conserved amino acids: pGln(Q)-Thr(T)-Phe(F)-Gln(Q)-Tyr(Y)-Ser(S)-Arg(R)-Gly(G)-Trp(W)-Thr(T)-Asn(N) (**Figure 4A**). The protein is mainly comprised of α-helices with some coiled structures (**Figures 4B, C**). We predicted a long helix region from sites 6 to 51 (**Figure S2B**). The protein model indicates a -2.71 C-score and a 0.40 ± 0.14 TM score, according to the I-TASSER analysis (**Figure 4D**). The Crz protein features four hydrophobic clusters (47.38 average residues), nine hydrogen bond networks (146.21° average DHA and 2.88 average distance between bonds), and 19 salt bridges (0.24 FCR and 0.21 value).

**Figure 5:**
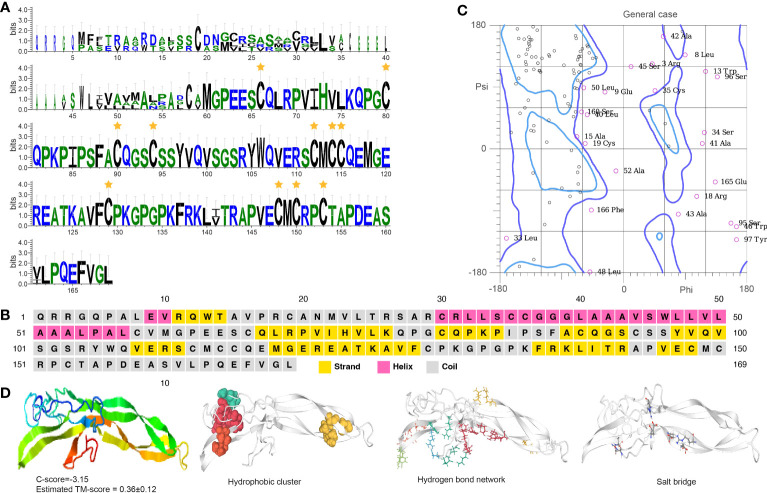
Functional domain and structural modeling of burs α in *A. americanum*. **(A)** Sequence logo analysis of the bursα protein. The sequence logo shows the conservation of amino acid residues at each position, with each letter’s height representing the conservation degree. The stars indicate the conserved cysteine domain. **(B)** Secondary structure prediction of burs α protein sequence using PSIPRED. **(C)** Ramachandran plot analysis for the crystal structure of burs α protein. The analysis indicates that most of the residues are in the allowed and generously allowed regions, suggesting the good quality of the protein structure. **(D)** I-TASSER analysis of the protein structure for burs α protein. The model consists of multiple domains with different colors representing different domains. The modeling protein structure was analyzed using ProteinTools to find hydrophobic clusters, hydrogen bond networks, and salt bridges for the EH protein.

**Figure 6:**
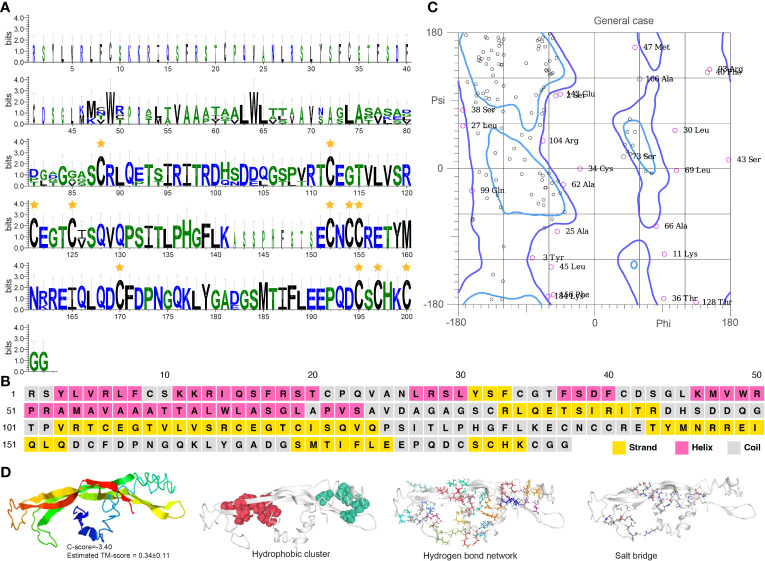
Functional domain and structural modeling of burs β in *A. americanum*. **(A)** Sequence logo analysis of the burs β protein. The sequence logo shows the conservation of amino acid residues at each position, with each letter’s height representing the conservation degree. The star indicates the conserved cysteine domain. **(B)** Secondary structure prediction of burs β protein sequence using PSIPRED. **(C)** Ramachandran plot analysis for the crystal structure of burs β protein. The analysis indicates that most of the residues are in the allowed and generously allowed regions, suggesting the good quality of the protein structure. **(D)** I-TASSER analysis of the protein structure for burs β protein. The model consists of multiple domains with different colors representing different domains. The modeling protein structure was analyzed using ProteinTools to find hydrophobic clusters, hydrogen bond networks, and salt bridges for the EH protein.

Burs α and burs β sequences feature Pfam domains PF00573 and PF03955, respectively. They have 11 cysteine residues located in specific positions within the protein, forming five disulfide bonds that likely contribute to protein stability and activity (**Figures 5A**, **6A**). Burs α has a predominantly α-helical secondary structure, with some regions of β-strands and turns (**Figures 5B, C**, **S2C**). The α-helices are arranged in a coiled-coil structure. Similarly, burs β was predicted to have a high percentage of α-helices, with scattered regions of β-strands and turns (**Figures 6B, C**, **S2D**). The overall structure of burs β was proposed to be a long helical bundle, which may be necessary for interacting with burs α and forming the active bursicon heterodimer. The I-TASSER results predicted that the C-score and TM-score for burs α (**Figure 5D**) and burs β (**Figure 6D**) were -3.15 and 0.36 ± 0.12, and -3.40 and 0.34 ± 0.11, respectively. Protein structures indicate to us that burs α and burs β have four (43.55 average residues) and two (44.90 average residues) hydrophobic clusters, nine (2.86 average distance and 152.43° average DHA) and 19 (2.81 average distance and 150.38° average DHA) hydrogen bond networks, and three (0.18 FCR and 0.21 κ value) and ten (0.21 FCR and 0.21 κ value) salt bridges.

### Gene structural and functional analyses

**Figure 7:**
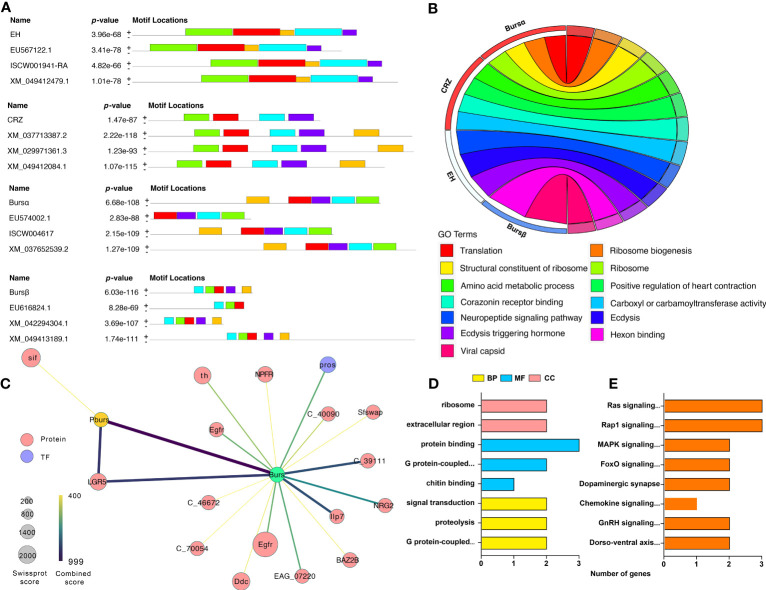
Gene structure, function, and protein interactive network of identified ERN genes. **(A)** Identification of conserved gene motifs in ERN genes utilizing the MEME tool. **Table S5** lists the specifics of the comparison species. **(B)** Co-expression of ERN genes and their functions. The chord’s color represents the associated functions. **(C)** A network of related ERNs and nodes that modulates. The STRING database and Cytoscape software were used to build the network. Each node represents a protein, and protein-protein interactions are represented by the edges linking the nodes. The node size represents the confidence score. The edge color represents the interactive score. For the created network, **(D, E)** GO, and KEGG Functional annotation analyses were performed.

MEME motif analysis indicates that the ERN genes have at least four to five patterns that are conserved with other tick species, such as *D. silvarum*, *R. sanguineus*, and *I. scapularis* (**Figures 7A**, **S3**). We analyzed the gene functions of the ERNs using GO analysis. All these genes are involved in ecdysis, including ecdysis-triggering hormone activity, neuropeptide signaling pathway, and Crz receptor binding (**Figure 7B**). Other biological processes, such as ribosome biogenesis, translation, and hexon binding, are also regulated by the identified ERNs.

We used the String database to reveal the connections between ERNs and other *A. americanum* proteins. The regulatory network indicates that only two ERNs (burs α and burs β) have direct interactions with 17 proteins (**Figure 7C**, **Table S5**), indicating that bursicon proteins are potentially involved in some signaling pathways and processes in *A. americanum*. One protein, LGR5 (leucine-rich repeat-containing G-protein coupled receptor 5), is highly connected to burs α and burs β. The functional annotation analysis shows that the whole network acts in multiple GO enrichment functions (e.g., chitin binding, G protein-coupled receptor activity, and signal transduction) and KEGG pathways (e.g., MAPK, gonadotropin-releasing hormone (GnRH), and chemokine signaling pathways) (**Figures 7D, E**).

### Developmental and tissue-specific expression profiles of ERNs

We ran RT-qPCR to validate the presence of the ERNs in four *A. americanum* tissues (midguts, synganglia, and salivary glands) (**Figure S4**). The *EH* expression was highest in the synganglia, with relatively low expression among salivary glands and midguts. Expression levels of the other three ERNs were also highest in the synganglia and with barely detectable levels in salivary glands and midguts, indicating that synganglia are the primary organs for the accumulation or synthesis of most ERNs.

Regarding developmental distribution, *EH*, *burs α*, and *burs β* have highest transcript levels in 0 h female adults, which significantly decreased over the following 24 h (**Figure S5**). *Burs α* is also highly expressed in 0 h larvae (**Figure S5**). *Crz* maintained expression across all developmental stages, with a peak in 0 h larvae and followed by nymphs, 0 h female adults, 24 h female adults, and eggs. Our interpretation is ERNs act in newly emerged larvae and female adults.

### *Burs α* and *burs β* RNAi efficiency and effects

**Figure 8:**
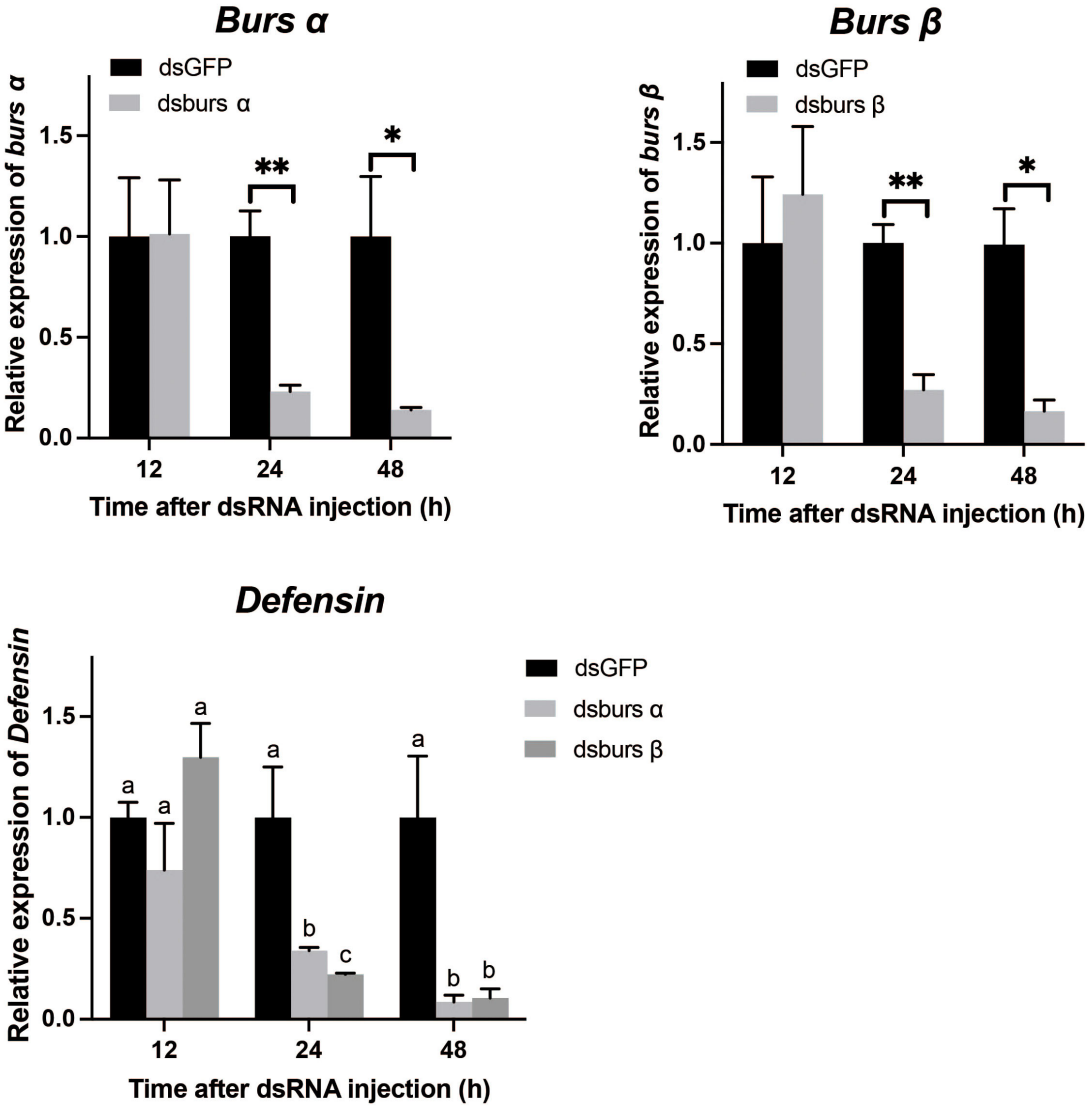
The efficiency of burs α and burs β RNAi and their effects on *Defensin* transcript levels. */** indicate significant differences (*p* < 0.05, 0.01). Different letters on the top of bars indicate that the means ± SEM are significantly different among treatments by *t*-test (*p* < 0.05).

Adults were treated with 1 μg dsRNA/tick, and then collected at 12, 24, and 48 h after injection. **Figure 8** shows the transcript levels of *burs α* were significantly decreased from 24 to 48 h post dsRNA injection. The RNAi efficiency increased from 76.9% at 24 h to 86.0% at 48 h, compared to controls. Similarly, *burs β* RNAi efficiency was 72.8% at 24 h and 83.4% at 48 h post-injection. Relative to controls, expression of an AMP gene, *Defensin* (34), was decreased by 66.0% and 91.7% at 24 and 48 h post dsBurs α treatment, respectively (**Figure 8**). Similarly, *Defensin* transcript levels were decreased by 77.8% and 89.6% at 24 and 48 h post dsBurs β injection, respectively (**Figure 8**).

## Discussion

Neuropeptides are produced by all animals with a nervous system, where they influence a wide range of biological functions. EH acts in all arthropods, regulating ecdysis in all developmental stages. A gene encoding EH was reported for *I. scapularis* and *R. microplus* (35, 36). Mature EH peptides have some common characteristics, such as the presence of six cysteine residues in conserved positions in each peptide chain, the formation of three intramolecular disulfide bonds, and varying lengths of signal peptides at the N-terminus of the mature peptide (28). In Araneae, the EH sequences of spiders and ticks have conserved cysteine binding sites, indicating the conservation of EH amino acid sequences. EH regulates changes in ecdysis and the release of tanning hormones in *Drosophila* larval ecdysis (37). Here, functional gene enrichment analysis shows that EH acts in the enrichment term “neuropeptide signaling pathway, ecdysis, and ecdysis-triggering hormone” in ticks. The expression of EH was higher in the *A. americanum* synganglia than in the other two tissues. Further research into the synthesis site and receptor proteins that transduce EH action will increase understanding of tick development and ecdysis. The presence of a tick ETH receptor is documented (38), although the gene sequences encoding the hormone have not been reported. Due to the absence of published gene sequences and the specific cellular sources of the hormone (38), we did not include the analysis of the ETH.

Crz is found in both insects and some crustaceans, with 11 highly conserved amino acids and the sequence of pGln-Thr-Phe-Gln-Tyr-Ser-Arg-Gly-Trp-Thr-Asn amide (39, 40). This is consistent among the ticks in this study. While the bioactive form of Crz is post-translationally cleaved from a precursor, the predicted structures inform us about potential cleavage sites and post-translational processing events. This may shed light on the maturation of bioactive peptides. As a member of the invertebrate Gonadotropin-releasing hormone (GnRH) family, Crz has the closest phylogenetic relationship with the GnRH and its receptors in crustaceans (41). Crz was identified in the American dog tick (*D. variabilis*) synganglion, with an N-terminal glutamine converted to pyroglutamate and alpha-amidation of the C-terminal glycine. This arrangement is a conserved sequence domain that may participate in triggering pre-ecdysis behavior (14, 42). Additionally, the C-terminal amidation signal is essential for the amidation of the Gly residue at the C-terminus of the mature Crz peptide, contributing to the biological activity and stability of the mature peptide. As a homologous analogue of vertebrate GnRH, its secretion is closely related to arthropod heart rate, body color changes, group behavior, and physiological rhythms (43, 44). Functional gene annotation analysis also shows that Crz may positively regulate heart contraction, consistent with evidence that Crz acts in regulating heartbeat and muscle contraction. Additionally, the high expression of Crz in 0-hour larvae suggests to us it is involved in the early stages of tick development. We suggest it may act in initiating and coordinating physiological processes required for larval growth and development (14, 15).

Arthropod molting is divided into three steps: splitting and shedding the old cuticle, extending and swelling of the new cuticle, and hardening and darkening the new cuticle (45). Cuticle hardening and darkening involve changes in cuticular proteins. Tanning hormones act in pigment deposition, such as melanin, pteridines, and quinones. After secretion, bursicon proteins promote the synthesis of cAMP, which leads to PKA activation, phosphorylation of tyrosine hydroxylase, and synthesis of tanning agents (17, 46). Our results indicate the homologous dimer formed by the polymerization of burs α or burs β subunits has a consistent disulfide bond structure in *A. americanum*. Previous reports document burs α and burs β transcripts in *R. microplus* (36), *D. variabilis* (42), *I. scapularis* (35), and *A. americanum* (47). In *A. americanum*, the closely associated node in the protein regulatory network is LGR5. This is consistent with previous studies reporting bursicon action is mediated by the GPCR and LGR families (48, 49). The protein network regulated by bursicon also functions in chitin binding, proteolysis, and signaling pathways (MAPK, Ras, RAP1, FOXO, dorso-ventral axis). We assessed the potential immune-related function of bursicon in *A. americanum* through RNAi, which revealed that inhibition of *burs α* and *burs β* resulted in a significant reduction in the expression of one AMP, Defensin. Our interpretation is that burs α and burs β act in signaling or regulatory pathways, controlling the expression of immune-related genes (19, 50). Understanding the immune-regulatory roles of neuropeptides like bursicon in ticks could significantly contribute to our knowledge of tick-host-pathogen interactions and open avenues for potential control strategies targeting tick-borne diseases.

Our structural modeling data may guide future studies. The predicted ERN three-dimensional structures provide valuable insights into their potential functions, which may be a basis for formulating hypotheses about the neuropeptides’ roles in *A. americanum*. The structural modeling data can shed light on the interactions between the ERNs and their respective receptors.

## Conclusion

Some tick species transmit diseases to humans. The Centers for Disease Control and Prevention reported over 50,000 U.S. cases of tickborne diseases, mostly Lyme disease, in the U.S. in 2019. Tickborne disease drives research interest in these animals. Here, our work on the biology of ERNs has potential for invention of entirely novel tick management technology based on silencing specific genes. These would include genes encoding the ERNs and their cell surface receptors. The work also identified genes encoding proteins the ERNs interact with, which are also potential targets.

## Data availability statement

The datasets presented in this study can be found in online repositories. The names of the repository/repositories and accession number(s) can be found below: https://www.ncbi.nlm.nih.gov/genbank/, PRJNA982785.

## Ethics statement

Ethical review and approval was not required for your study of ticks in accordance with local legislation and institutional requirements. Animal welfare of arthropods used in research and teaching are not covered by federal, state, or local agencies.

## Author contributions

QS: conceptualization, methodology, reviewing. BL: writing original draft, data analysis, and visualization. JL: writing original draft, data collection and investigation. BN: data collection, reviewing, investigation. DS: supervision, editing, and reviewing. All authors contributed to the article and approved the submitted version.
