# Supplementary material for: Identification and characterization of ecdysis-related neuropeptides in the lone star tick Amblyomma americanum
Source: Front Endocrinol (Lausanne). 2023 Aug 25;14:1256618. doi: 10.3389/fendo.2023.1256618 (PMC10490126; doi:10.3389/fendo.2023.1256618)
Supplement: Supplementary file 2 [file DataSheet_2.docx]

**Identification and characterization of ecdysis-related neuropeptides in the lone star tick *Amblyomma americanum***

Bo Lyu^a,1^, Jingjing Li^a,1^, Brigid Niemeyer^a^, David Stanley^b^, Qisheng Song^a,*^

^a^Division of Plant Science and Technology, University of Missouri, Columbia, MO 65211, USA

^b^Biological Control of Insect Research Laboratory, United States Department of Agriculture-Agricultural Research Station (USDA/ARS), Columbia, MO 65203, USA

^1^ Co-first authors

^*^Corresponding author

Qisheng Song; E-mail: SongQ@missouri.edu; Phone number: +1 5738829798;


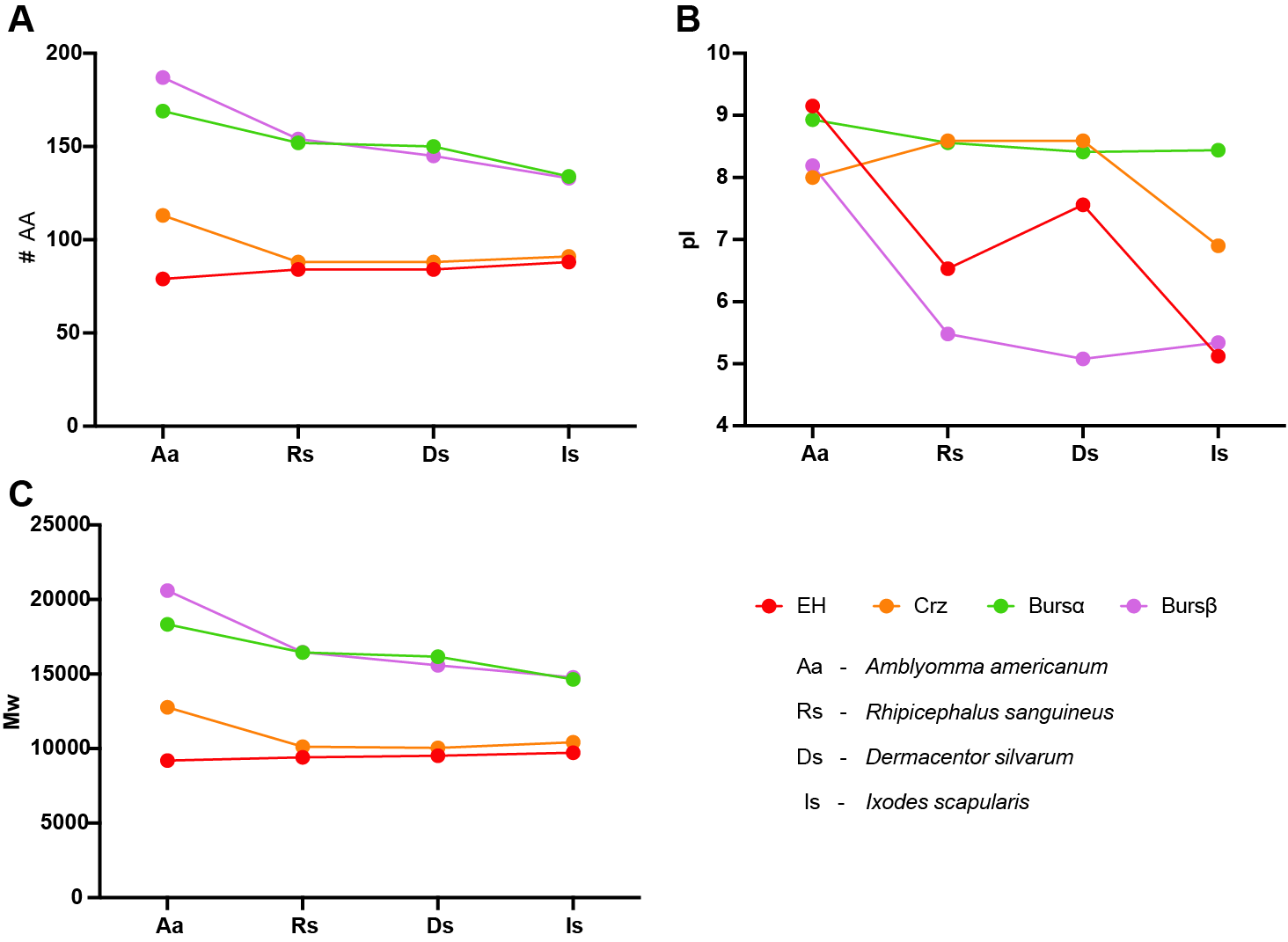


Figure S1. Comparison of amino acid length (A), isoelectric points (B), and molecular weights (C) in tick species.


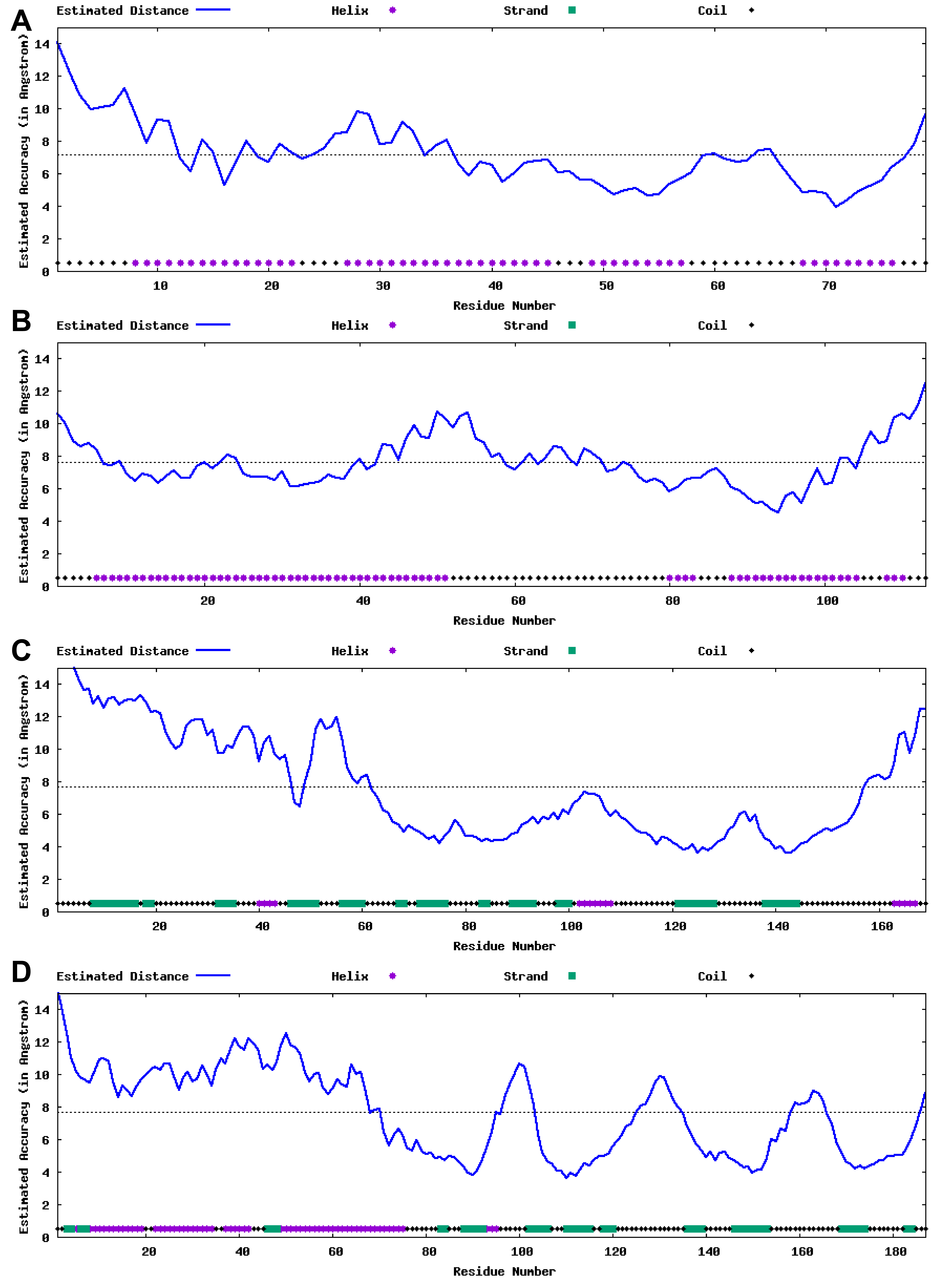


Figure S2. The predicted normalized B-factors obtained from the I-TASSER protein structure prediction method. Normalized B-factors provide insights into the flexibility or rigidity of ERNs regions within the predicted models.


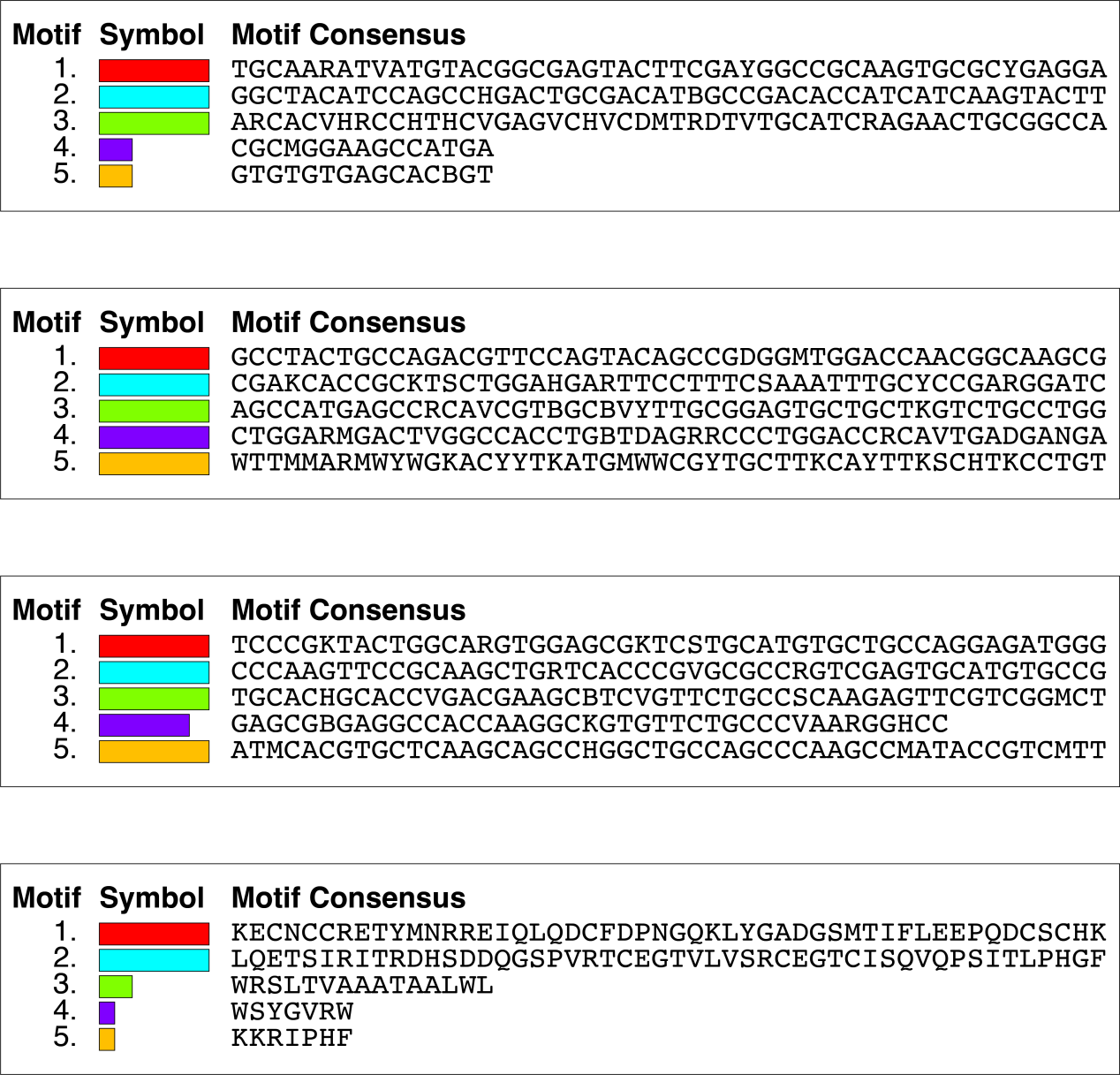


Figure S3. Top five motif consensus of the identified ERNs genes in *Amblyomma americanum*.


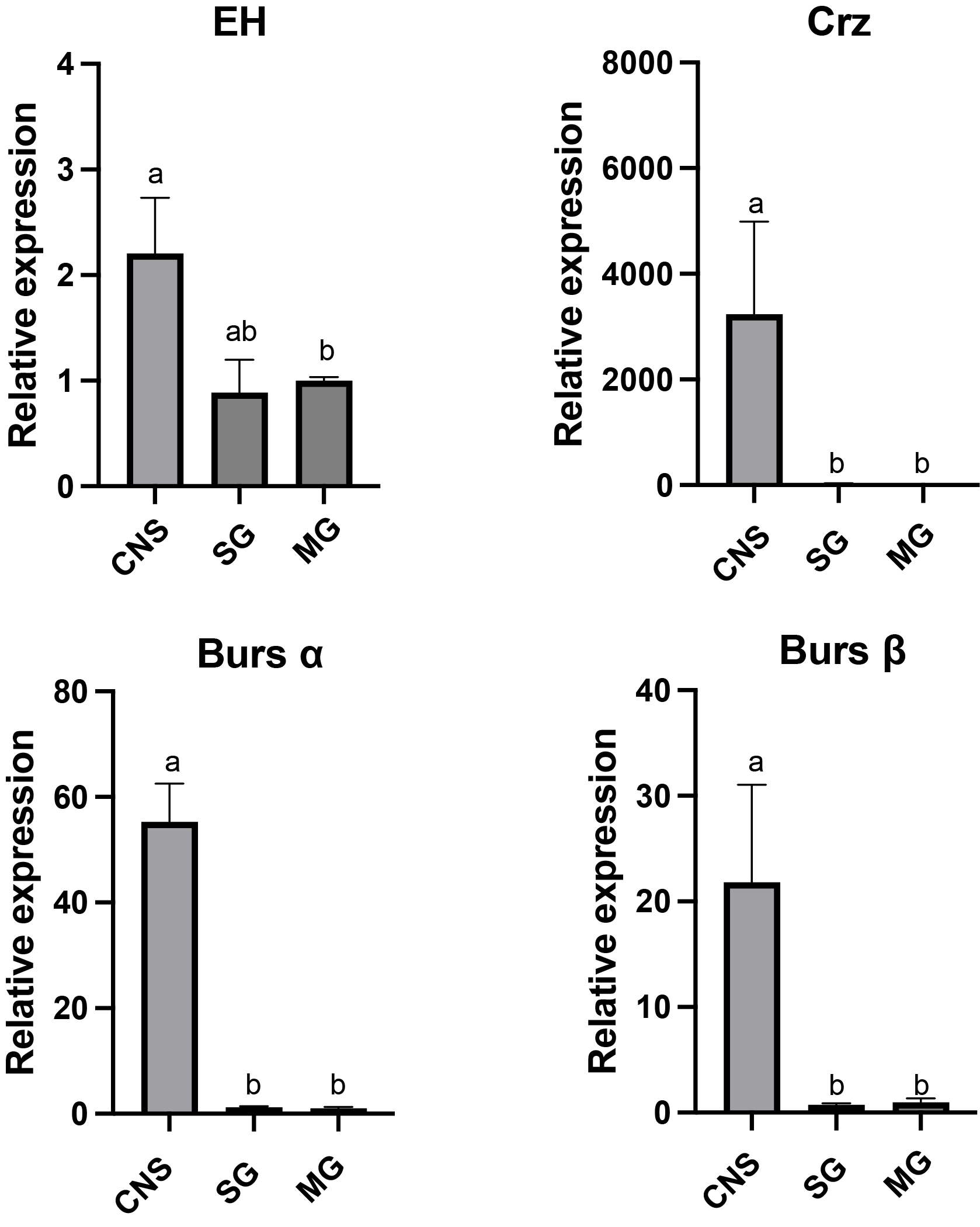


Figure S4. Tissue specific analysis of ERN genes using qRT-PCR. The gene and primer details are listed in Table S1. CNS indicaates central nerve system (synganglia), SG represents salivary glands, and MG represents midgut. ^ab^ different letters indicate significant differences (*p* < 0.05).


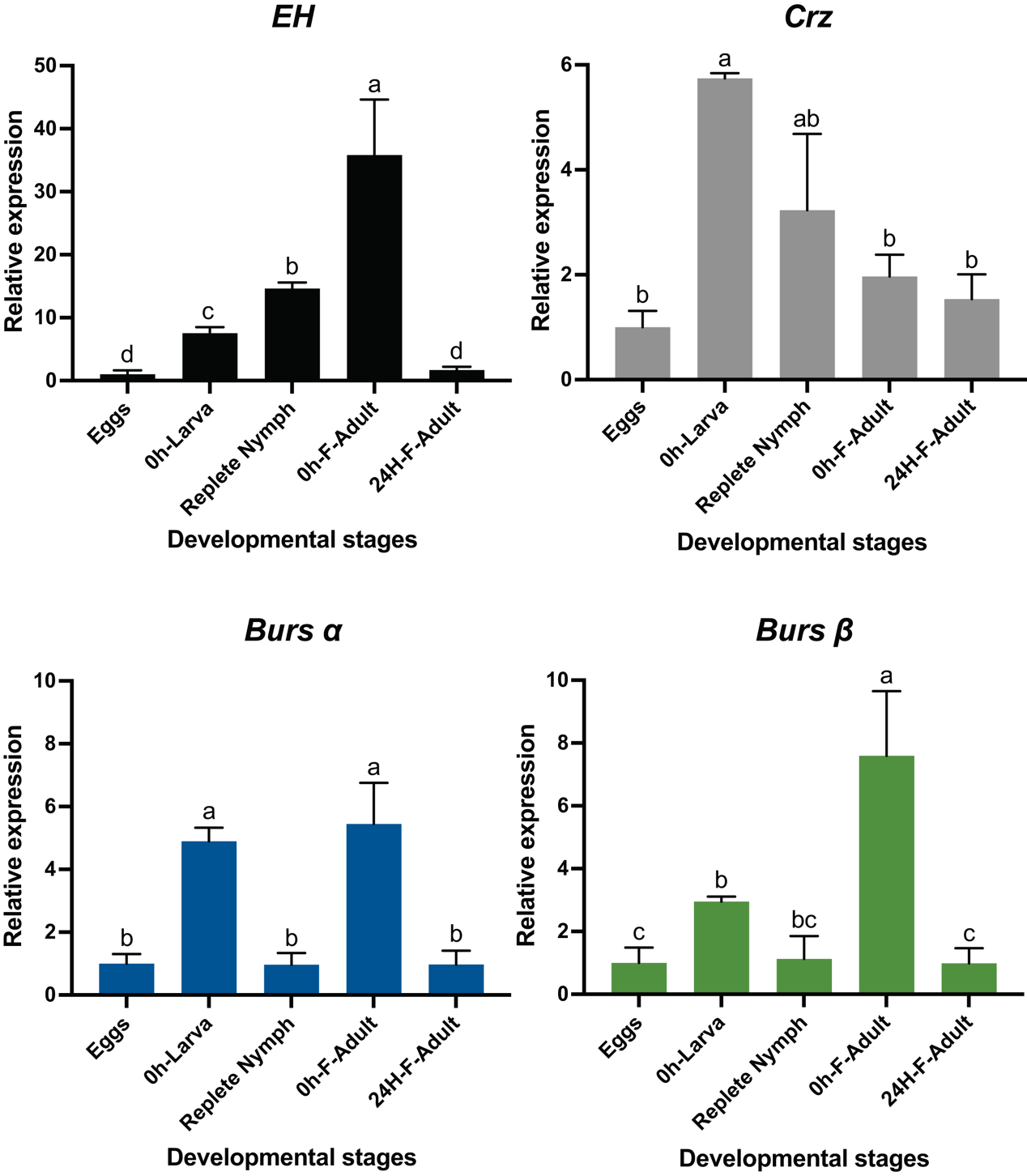


Figure S5. Developmental specific analysis of ERN gene expressions using qRT-PCR. Different letters on the top of bars indicate that the means ± SEM are significantly different among treatments by t-test (*p* < 0.05).
